# An Epidemiology‐Constrained Framework for Mechanistic Hypothesis Restriction and Prioritization in Complex Disease

**DOI:** 10.1111/jep.70638

**Published:** 2026-10-06

**Authors:** Uri Gabbay

**Affiliations:** ^1^ Gray Faculty of Medicine and Health Sciences Tel Aviv University Tel Aviv Israel

**Keywords:** causal inference, complex disease, epidemiology, mechanistic inference, multimorbidity, philosophy of medicine, systems medicine

## Abstract

**Rationale:**

Many chronic diseases remain mechanistically unresolved despite major advances in molecular biology, systems biology and high‐throughput biomedical investigation. Disorders such as autoimmune disease, neurodegeneration and chronic inflammatory syndromes frequently exhibit prolonged latency, developmental susceptibility windows, heterogeneous phenotypes and multiscale biological interaction that limit direct experimental reconstruction of initiating causal events. Under such conditions, causal interpretation may proceed without sufficient inferential constraint, contributing to fragmentation between molecular association and coherent causal explanation.

**Aims and Objectives:**

To propose an epidemiology‐constrained framework for restricting and prioritising mechanistic hypotheses in complex chronic disease when direct experimental access to disease initiation is limited.

**Methods:**

This conceptual framework was developed through a purposive conceptual synthesis of recurring epidemiologic structures and established inferential approaches relevant to complex chronic disease. The literature was organised across predefined conceptual domains, including temporality, sex distribution, geographic and migration patterns, multimorbidity, protective exposures, life‐course epidemiology, causal inference, triangulation and systems epidemiology. Multiple sclerosis and Parkinson's disease were selected as illustrative examples because they exhibit several characteristics central to the framework, including prolonged latency, environmental modulation, heterogeneous phenotypes and limited experimental accessibility to initiating events.

**Results:**

Within the proposed model, structured epidemiologic observations, including sex distribution, geographic gradients, migration effects, age‐of‐onset patterns, multimorbidity networks and protective exposures, are first evaluated for reproducibility, bias, temporality and alternative explanations and are then treated as candidate constraints on competing mechanistic models. These constraints restrict and prioritise, rather than establish mechanistic hypotheses. Temporality and multimorbidity are incorporated as inferential filters that help distinguish antecedent causal processes from downstream consequences and treatment‐related effects. Application to multiple sclerosis and Parkinson's disease illustrates how convergent population‐level observations may narrow mechanistic hypothesis space prior to molecular validation.

**Conclusions:**

Epidemiologic structure may serve not merely as descriptive association but as inferential architecture for systematically restricting and prioritising mechanistic hypotheses under conditions of limited experimental accessibility. Epidemiology‐constrained inference does not establish mechanisms or causality independently, but may provide a complementary framework for organising, comparing and prioritising competing mechanistic models.

## 1 Introduction

Despite unprecedented advances in molecular biology, many chronic diseases remain mechanistically unresolved. We use the term complex chronic disease to describe chronic disorders characterised by prolonged or heterogeneous disease trajectories, multiscale interactions among genetic, environmental, developmental and biological processes, limited experimental accessibility to initiating events, phenotypic heterogeneity and reproducible population‐level structure. Mechanistic uncertainty is treated here as a common consequence of these characteristics rather than as a defining criterion [1, 2, 3, 4, 5].

The novelty of the proposed framework does not lie in any individual epidemiologic method. Temporality, migration studies, natural experiments, triangulation, causal graphs, and life‐course approaches are established components of contemporary epidemiology. Rather, the framework integrates these traditions around a specific inferential function: reproducible and bias‐assessed epidemiologic structure is treated as an explicit constraint on competing mechanistic models before and during molecular investigation. The proposed contribution is therefore a structured sequence linking population‐level observations to mechanistic hypothesis restriction and experimental prioritization.

This discrepancy reflects an important epistemic challenge in contemporary biomedical science. Although contemporary epidemiology provides robust tools for identifying associations and estimating causal effects, less attention has been devoted to how population‐level structure may constrain the space of biologically plausible mechanisms. Molecular reductionism is highly effective once causal architecture is approximately known. However, in diseases characterised by long preclinical phases, developmental susceptibility windows and multifactorial causation, the initiating causal processes may precede diagnosis by years or decades and may no longer be experimentally accessible at the time disease becomes clinically apparent [1, 2, 3, 4, 5, 6].

Molecular pathways may become statistically associated with disease while remaining uncertain regarding causal position, temporal ordering, or mechanistic centrality.

We propose that epidemiologic structure itself may provide an underutilized source of mechanistic constraint. Population‐level patterns, including sex bias, geographic gradients, migration effects, age‐of‐onset distributions, multimorbidity clustering and protective exposures, may function as boundary conditions restricting the range of plausible causal explanations before experimental investigation proceeds.

This article proposes an epidemiology‐constrained framework for mechanistic inference in complex chronic disease. In this article, ‘mechanistic inference’ refers specifically to the structured restriction and prioritization of competing mechanistic hypotheses based on epidemiologic constraints; it does not imply identification of a unique mechanism or independent establishment of causality. The framework does not replace molecular biology, translational experimentation, or established epidemiologic methods. Rather, it seeks to organise mechanistic reasoning when initiating causal events cannot be directly reconstructed experimentally.

## 2 Conceptual Approach

This article presents a conceptual framework developed through purposive conceptual synthesis rather than systematic review or empirical analysis. The literature was selected to represent established inferential domains relevant to mechanistic reasoning under limited experimental accessibility, including temporality, sex distribution, geographic and migration patterns, age‐of‐onset structure, multimorbidity, protective exposures, life‐course epidemiology, causal inference, triangulation, systems epidemiology and exposome research. These domains were examined for their potential to provide reproducible constraints on competing mechanistic models.

The primary unit of analysis is the candidate mechanistic model. Epidemiologic observations constitute potential constraints on that model rather than direct evidence of a specific biological mechanism. A constraint is considered informative when the observation is reproducible, temporally relevant, reasonably robust to major sources of bias, and capable of differentiating among competing mechanistic explanations. The framework therefore distinguishes between an epidemiologic observation, its validation as a constraint, and the mechanistic hypothesis that remains compatible with that constraint.

The framework was developed iteratively by integrating these domains into a common inferential sequence: observation, constraint validation, compatibility assessment, hypothesis restriction and experimental prioritization. Multiple sclerosis and Parkinson's disease were selected as illustrative examples because they exhibit several epidemiologic characteristics central to the framework, including prolonged latency, environmental modulation, heterogeneous phenotypes and limited experimental accessibility to initiating events.

## 3 The Problem of Limited Experimental Accessibility

Many complex diseases exhibit prolonged preclinical phases during which causal processes evolve silently before overt disease expression. Autoantibodies may precede autoimmune disease by years, neurodegenerative changes may accumulate long before symptom onset, and developmental or environmental exposures may exert effects during temporally sensitive periods that cannot later be experimentally reconstructed [1, 2, 3, 4, 5].

In these settings, randomised experimentation is frequently infeasible or ethically impossible. Early‐life environmental exposures, migration history, sex‐specific developmental biology and long‐term host‐environment interaction cannot readily be manipulated experimentally in humans [3, 6, 7, 8].

This creates a condition of limited experimental accessibility in which the initiating causal events may be only indirectly inferable from later population structure. Under such circumstances, causal reasoning often depends upon convergent epidemiologic observations rather than direct manipulation of initiating biology. In such settings, mechanistic inference requires a form of causal reconstruction that differs from classical laboratory experimentation, requiring integration of indirect but convergent signals across biological and population scales. Population‐level structure therefore becomes not merely descriptive but potentially informative regarding permissible mechanisms.

## 4 Epidemiology as Inferential Architecture

In experimentally accessible systems, mechanistic hypotheses may be directly tested through intervention and controlled manipulation. In complex chronic disease, however, hypothesis generation assumes an additional epistemic role. It functions as an inferential bridge linking structured population observations with biologically plausible mechanistic models.

Within this framework, mechanistic hypothesis restriction proceeds through five sequential steps:

1. Identification and, where feasible, prespecification of reproducible epidemiologic observations. Where feasible, candidate constraints should be specified before mechanistic testing to reduce selective interpretation of observations after a favoured mechanism has emerged. The initial constraint set should include both potentially supportive and potentially disconfirming epidemiologic observations.
2. Validate candidate observations by assessing temporality, reproducibility, confounding, selection, ascertainment, reverse causation, treatment effects and plausible non‐biological explanations.
3. Mapping validated constraints against competing mechanistic models. Each competing model should, where feasible, be evaluated against the same prespecified constraint set, including observations that could potentially disconfirm the model.
4. Evaluate the degree of convergence across partially independent constraints, explicitly accounting for correlated observations and conflicting evidence.
5. Prioritization of mechanistic models for experimental or translational testing, with explicit consideration of disconfirming evidence and independent replication where available.

For operational purposes, ‘compatible’ denotes that a candidate mechanism can plausibly generate or accommodate the observed epidemiologic structure without requiring major unsupported assumptions; ‘incompatible’ denotes that the mechanism conflicts with a well‐established temporal, population, or phenotypic constraint; and ‘uncertain’ denotes insufficient evidence to distinguish compatibility from incompatibility. These classifications are intended as structured judgements rather than quantitative scores unless empirical evidence permits formal weighting.

The framework does not require a universal numerical threshold for mechanistic deprioritization, because the evidentiary meaning of a constraint is dependent on the disease, population, study design and quality of the underlying evidence. Instead, a candidate model should be deprioritized when one or more validated constraints are difficult to accommodate without introducing additional unsupported assumptions, particularly when the incompatibility is reproducible across independent populations or arises from partially independent constraint domains. The strength of deprioritization should therefore reflect the reproducibility, independence, validity and degree of incompatibility of the relevant constraints rather than their simple numerical count. When competing models remain similarly compatible with the validated constraint structure, the appropriate output is retained uncertainty rather than forced ranking.

This mode of reasoning relies upon convergent constraints rather than direct experimental reconstruction of initiating events. Accordingly, epidemiologic observations are treated not solely as associations but as components of inferential architecture that may help delimit the plausible hypothesis space. A proposed mechanism may therefore lose plausibility not only through failed experimental validation, but also through incompatibility with reproducible population‐level structure.

## 5 Validation of Epidemiologic Constraints

Not every epidemiologic association qualifies as a mechanistic constraint. Before an observation is incorporated into the framework, its potential inferential value should be evaluated against alternative explanations and major sources of bias. These include residual confounding, selection and ascertainment bias, diagnostic instability, differential healthcare utilization, treatment effects, socioeconomic differences, exposure misclassification and reverse causation.

The framework therefore distinguishes three levels of interpretation: (1) an observed epidemiologic pattern; (2) a validated epidemiologic constraint that remains reproducible after plausible alternative explanations have been considered and (3) a mechanistic implication that is compatible with the validated constraint. This distinction prevents direct movement from population association to biological explanation.

Constraints should also be evaluated for informational independence. Multiple observations should not be treated as equivalent independent evidence merely because they are statistically or descriptively distinct. Geographic gradients, migration effects, environmental exposures and early‐life timing, for example, may reflect overlapping causal processes. Inferential strength therefore depends not simply on the number of observations but on the extent to which they provide partially independent or orthogonal constraints on competing models.

When epidemiologic constraints conflict, the framework does not require selection of a preferred mechanism solely on the basis of numerical accumulation. Conflicting constraints should instead trigger reassessment of measurement, bias, temporal structure, population heterogeneity and model specification. An unresolved conflict is therefore retained as uncertainty rather than converted into a mechanistic conclusion. Conflicting constraints should therefore be treated as information about model inadequacy, population heterogeneity, measurement uncertainty, or incomplete specification rather than as evidence to be resolved by majority counting. A model should not be prioritised solely because it accommodates a larger number of correlated observations.

## 6 Epidemiologic Constraints as Boundary Conditions

Certain epidemiologic observations may function as candidate constraints on mechanistic explanation when they are reproducible, temporally informative and sufficiently robust to major alternative explanations.

### 6.1 Sex Distribution

Many autoimmune diseases exhibit marked female predominance [9, 10, 11, 12, 13, 14]. Such patterns may constrain candidate mechanisms toward explanations capable of accommodating sex‐linked immune regulation, hormonal modulation, epigenetic divergence or X‐chromosome‐associated biology. However, female predominance should not itself be interpreted as evidence for a biological mechanism until alternative explanations, including differential exposure, diagnosis, healthcare utilization and ascertainment, have been considered. The resulting constraint is therefore conditional: mechanisms that cannot plausibly accommodate the reproducible sex distribution may be deprioritized, whereas several biologically distinct mechanisms may remain compatible. Such asymmetry may be difficult to explain solely as a downstream consequence of established pathology.

### 6.2 Geographic Gradients

Latitude‐associated gradients observed in diseases such as multiple sclerosis suggest environmental or photobiologic influences linked to geography, infectious ecology, circadian biology or vitamin D metabolism [15, 16, 17, 18, 19]. These gradients frequently emerge early in life, supporting developmental environmental modulation.

### 6.3 Migration Effects

Migration studies can provide quasi‐experimental information regarding environmental modification of disease risk [20, 21, 22]. Risk convergence among migrant populations may weaken purely genetic explanations when migration is followed by reproducible changes in disease risk. However, migration effects may also reflect selection, acculturation, socioeconomic change, healthcare access, diagnostic differences or intergenerational effects. Migration therefore functions as a mechanistic constraint only after these alternative explanations have been evaluated.

### 6.4 Age‐of‐Onset Structure

Age distributions may also constrain mechanistic interpretation. Paediatric‐onset disease suggests developmental susceptibility, reproductive‐age peaks imply hormonal interaction, and late‐onset disorders may reflect immunosenescence, cumulative exposure burden, or metabolic decline [23, 24].

Collectively, these observations may help constrain the range of biologically plausible causal explanations. Table 1 summarises examples of epidemiologic observations and the corresponding mechanistic constraints they may impose.

Importantly, no individual epidemiologic observation uniquely determines mechanism; inferential strength instead emerges through convergence across multiple constraints that provide, where possible, partially independent or orthogonal information. The number of observations should not be equated with the strength of inference; correlated observations may provide little additional information, whereas partially independent constraints may substantially narrow the space of compatible models.

## 7 Temporality and Multimorbidity in Mechanistic Inference

### 7.1 Temporality as an Inferential Filter

Temporality remains among the strongest discriminators between antecedent causal processes and downstream consequences [25]. Observations that consistently precede clinical diagnosis may provide temporal constraints, but temporal precedence alone does not establish causal priority. In diseases with prolonged subclinical phases, biomarkers, symptoms or comorbidities that precede diagnosis may already represent early manifestations of disease biology. Temporality should therefore be interpreted jointly with evidence regarding disease onset, reverse causation, and the plausibility of prodromal processes. Accordingly, temporality functions as an inferential filter rather than a causal criterion.

### 7.2 Multimorbidity as Mechanistic Signal

Multimorbidity is often treated analytically as confounding or statistical complexity. However, structured comorbidity patterns may also provide insight into shared upstream dysregulation [26, 27, 28].

Examples include:

- clustering among autoimmune diseases,
- associations between inflammatory disorders and chronic fatigue syndromes,
- metabolic dysregulation accompanying neurodegenerative disease.

When temporally antecedent or independent of disease severity, such associations may indicate shared regulatory disturbances rather than unrelated secondary phenomena, particularly when such clustering persists after accounting for healthcare utilization, treatment exposure, and demographic confounding. This logic is compatible with causal graph frameworks in which temporally antecedent variables constrain permissible causal directionality.

Distinguishing antecedent multimorbidity from treatment effects or downstream disease consequences therefore becomes essential for mechanistic interpretation.

Structured multimorbidity should therefore be treated as a candidate constraint rather than a mechanistic signal by default. Its inferential value increases when clustering is reproducible across independent populations, persists after accounting for healthcare utilization, treatment exposure, age, socioeconomic position and other major confounders, and demonstrates temporal or biological coherence across related phenotypes. Conversely, clustering that disappears after such adjustment should not be used to restrict mechanistic models.

## 8 Protective Factors and Resilience Biology

Protective exposures may illuminate mechanism as clearly as risk factors. Early‐life microbial diversity, environmental heterogeneity, pregnancy‐associated immune recalibration, and resilient phenotypes suggest that disease susceptibility is biologically modifiable rather than genetically deterministic [29, 30, 31].

Individuals exposed to substantial environmental or genetic risk who nevertheless remain disease‐free may represent informative models of regulatory protection. However, apparent resilience may arise through several distinct processes, including genuine protective biology, absence of the relevant susceptibility factor, exposure misclassification, competing risks, differential survival, or unmeasured protective exposures. Accordingly, resilient phenotypes should not be interpreted as evidence of a specific protective mechanism without first distinguishing these alternatives. The inferential value of resilience is greatest when protection is reproducible, exposure and susceptibility are well characterised, and candidate protective mechanisms can be contrasted with plausible selection or measurement explanations. Studying such resilient populations may help identify stabilising biological systems that prevent transition from susceptibility to pathology.

This perspective expands mechanistic investigation beyond pathology‐centred models toward resilience biology and regulatory maintenance. From a translational perspective, resilience‐associated mechanisms may prove more therapeutically informative than end‐stage pathological signatures.

## 9 Relationship to Reductionism and Systems Medicine

The proposed framework does not reject molecular reductionism; rather, it attempts to situate reductionist investigation within broader systems‐level causal reasoning.

Reductionist biology has transformed medicine by identifying molecular pathways, signalling networks and therapeutic targets. However, in diseases characterised by prolonged latency, developmental susceptibility and multiscale interaction, unconstrained molecular exploration may generate mechanistic fragmentation in which numerous associated pathways coexist without coherent causal integration.

Systems medicine has increasingly emphasised that chronic disease emerges through interaction between genetics, environment, immune regulation, metabolism, neurobiology and developmental timing [32, 33, 34, 35]. Within such models, disease emerges not from isolated pathways alone but from dysregulated interaction across biologic scales and time. The present framework extends this perspective by proposing that epidemiologic structure may help prioritise among competing mechanistic models before large‐scale experimental investigation proceeds.

Rather than asking only which molecular pathways are statistically associated with disease, epidemiology‐constrained reasoning asks which mechanisms remain compatible with the total observed population structure.

This distinction may be particularly relevant in complex chronic diseases where direct reconstruction of initiating events is limited. The framework therefore occupies an intermediate inferential position between descriptive epidemiology and experimentally validated mechanistic biology.

## 10 Relationship to Existing Epidemiologic Frameworks

The proposed framework complements rather than replaces established approaches to causal inference.

Bradford Hill considerations remain central for evaluating causal plausibility [25], while triangulation approaches strengthen inference through convergence across study designs [36]. Life‐course epidemiology, exposome research and systems epidemiology similarly emphasise interaction between biology, environment and temporality [32, 33, 34, 35].

The present framework differs primarily in emphasising epidemiologic observations as mechanistic boundary conditions under circumstances in which direct experimental access to initiating causal events is limited. Unlike triangulation, which primarily strengthens causal inference through convergence across methodologies, the present framework emphasises convergence across epidemiologic structure as a means of constraining mechanistic plausibility. Similarly, whereas directed acyclic graph approaches primarily formalise causal assumptions among measured variables, the present framework addresses how large‐scale population structure may constrain upstream mechanistic interpretation. Rather than asking only which mechanisms are associated with disease, the framework asks which mechanistic explanations remain compatible with the integrated epidemiologic structure, as summarised in Table 2.

**Table 1:** Epidemiologic observations and candidate mechanistic model requirements.

Domain	Epidemiologic observation	Candidate mechanistic model requirement
Sex biology	Female predominance	Model should accommodate reproducible sex‐related heterogeneity
Environmental modulation	Migration risk convergence	Model should accommodate environmentally modifiable susceptibility
Developmental timing	Early‐life latitude effect	Model should accommodate developmental or early‐life environmental timing
Temporality	Stable preclinical autoantibodies	Model should accommodate prolonged preclinical biological processes
Systems regulation	Structured autoimmune clustering	Model should accommodate shared or interacting upstream processes
Prodromal structure	Prodromal non‐motor neurodegenerative symptoms	Model should accommodate an extended preclinical phase without assuming causal direction

**Table 2:** Comparison of epidemiology‐constrained mechanistic hypothesis restriction with related inferential frameworks.

Framework	Primary inferential target	Unit of analysis	Role of temporality	Role of mechanisms	Bias/confounding	Distinct contribution
Bradford Hill considerations	Causal plausibility	Exposure–outcome relationship	One causal consideration	Mechanistic coherence among considerations	Considered qualitatively	Structured causal appraisal
Triangulation	Causal inference	Exposure–outcome relationship across designs	Depends on design	Mechanisms inform interpretation	Exploits differing bias structures	Convergence across designs with different biases
DAG‐based inference	Causal identification/assumption specification	Variables and causal relations	Explicitly represented	Mechanistic assumptions may inform graph structure	Formal confounding/control structure	Explicit causal assumptions
Life‐course epidemiology	Timing and accumulation of exposures	Exposure trajectories/populations	Central	Biological embedding over time	Context‐dependent	Developmental and temporal organization
Exposome research	Comprehensive exposure characterization	Individual/population exposure profiles	Time‐varying	Links exposure mixtures to biology	Measurement and exposure bias	Integrated exposure characterization
Systems epidemiology	Multilevel population‐health relationships	Networks/populations	Multiscale	Integrates biological and population systems	Multilevel	Systems‐level organization
Epidemiology‐constrained mechanistic hypothesis restriction	Restriction and prioritization of competing mechanistic models	Candidate mechanistic model evaluated against epidemiologic constraints	Central inferential filter	Explicit target of hypothesis restriction	Constraint admitted only after bias/alternative‐explanation assessment	Uses convergent population structure as boundary conditions for mechanistic hypothesis space

## 11 Worked Example: Multiple Sclerosis

Multiple sclerosis (MS) illustrates how epidemiologic structure may constrain mechanistic inference.

Several epidemiologic observations consistently characterise MS:

- female predominance,
- latitude‐associated incidence gradients,
- migration‐dependent risk modification,
- young‐adult age‐of‐onset peak,
- interaction with environmental and infectious exposures [15, 16, 17, 18, 19, 20, 21, 22, 23, 24].

Individually, each observation may permit multiple interpretations. Collectively, however, they substantially narrow plausible mechanistic space. Taken together, these observations do not identify a unique mechanism, but they alter the relative compatibility of competing models. A purely stochastic model provides no obvious account for several reproducible population‐level constraints, whereas an exclusively cell‐autonomous model must additionally accommodate migration‐associated risk modification and geographic structure. An environmentally modulated immune model is therefore prioritised because it can accommodate a larger proportion of the integrated constraint structure.

Female predominance is compatible with sex‐linked immune regulation and hormonal modulation, among other possible explanations [9, 10, 11, 12, 13, 14]. Latitude gradients may support environmental or photobiologic influences operating during developmental windows [15, 16, 17, 18, 19]. Migration studies demonstrate environmentally modifiable susceptibility, arguing against purely genetic causation [20, 21, 22]. The characteristic age‐of‐onset distribution further supports interaction between developmental immune programming and later environmental triggers [23, 24].

The prioritization is therefore comparative rather than absolute: a model may be ranked below another model without being rejected, particularly when the same epidemiologic structure could plausibly arise from more than one biological architecture.

Together, these observations constrain mechanistic hypotheses toward environmentally modulated immune dysregulation acting upon genetically susceptible individuals during temporally sensitive developmental periods.

Molecular investigation may subsequently refine these mechanisms, but epidemiologic structure narrows the range of biologically plausible explanations before experimentation proceeds. The comparative constraint matrix illustrating this hypothesis‐restriction process is presented in Table 3.

**Table 3:** Illustrative constraint matrix for multiple sclerosis.

Epidemiologic constraint	Primarily stochastic model	Predominantly cell‐autonomous model	Environmentally modulated immune model
Female predominance	Uncertain/weak compatibility	Partial compatibility	Compatible
Latitude‐associated gradient	Uncertain	Weak compatibility	Compatible
Migration‐associated risk modification	Weak compatibility	Weak compatibility	Compatible
Young‐adult age‐of‐onset	Uncertain	Partial compatibility	Compatible
Environmental/infectious associations	Uncertain	Partial compatibility	Compatible
Overall assessment	Deprioritized	Not excluded	Prioritised for testing

*Note:* Compatibility judgements in this illustrative matrix are qualitative and are intended to demonstrate the logic of comparative hypothesis restriction rather than provide quantitative likelihood estimates. ‘Compatible’ indicates that a model can accommodate the constraint without major additional assumptions; ‘weak’ or ‘partial compatibility’ indicates that accommodation requires additional assumptions or leaves important aspects unexplained; ‘uncertain’ indicates that the available epidemiologic evidence does not permit a meaningful compatibility judgement. ‘Prioritised for testing’ does not imply causal confirmation.

## 12 Worked Example: Parkinson's Disease

Parkinson's disease (PD) provides an additional illustration of epidemiology‐constrained inference in neurodegenerative disease.

Several epidemiologic and clinical observations consistently characterise PD:

- prolonged prodromal phases,
- early non‐motor manifestations including constipation and hyposmia,
- age‐associated incidence increase,
- environmental associations including pesticide exposure,
- selective vulnerability of dopaminergic neurons,
- substantial clinical heterogeneity.

For illustration, these observations can be evaluated against at least three broad mechanistic models: (1) predominantly cell‐autonomous neurodegeneration, (2) generalised age‐related neurodegenerative susceptibility and (3) a multistage model involving systemic or peripheral processes, environmental exposures, host susceptibility and selective neuronal vulnerability. The epidemiologic structure does not uniquely distinguish among these models, but it creates differential compatibility. Prolonged prodromal manifestations are compatible with a multistage process, while selective neuronal vulnerability places constraints on explanations based solely on generalised aging. Environmental associations further support consideration of host–environment interaction, although they remain vulnerable to confounding and exposure misclassification.

Importantly, constipation and hyposmia should not automatically be interpreted as antecedent causal exposures. Because they may represent prodromal manifestations of PD, their principal contribution within this framework is to constrain temporal models of disease initiation and progression rather than to establish an upstream causal pathway.

These observations may constrain mechanistic interpretation in several ways.

Prodromal gastrointestinal and olfactory manifestations preceding motor disease are compatible with models in which pathophysiologic processes extend beyond the classical motor system, but do not by themselves establish that peripheral processes initiate disease. Environmental associations support interaction between host susceptibility and long‐term exposure biology rather than purely stochastic neurodegeneration. Selective neuronal vulnerability suggests that generalised aging alone incompletely explains disease localization.

Similarly, marked heterogeneity in progression and phenotype raises the possibility that Parkinson's disease may represent a syndrome encompassing partially distinct mechanistic pathways rather than a singular biologic entity. This possibility may help explain why molecular studies in PD frequently identify partially overlapping but non‐identical biological signatures across patient populations.

## 13 Operational Implications for Study Design

An epidemiology‐constrained framework has implications for study design and analytical strategy. Table 4 summarises how different types of epidemiologic constraints may be linked to study designs capable of evaluating their principal alternative explanations.

**Table 4:** Linking epidemiologic constraints to study design.

Constraint type	Illustrative question	Preferred methodological strategy
Migration structure	Does environmental context modify inherited susceptibility?	Migration/natural‐experiment designs
Early‐life timing	Does exposure during a developmental window alter later risk?	Life‐course longitudinal cohorts
Multimorbidity clustering	Do disease combinations indicate shared upstream processes?	Longitudinal multimorbidity/network analysis
Suspected mediation	Does an intermediate biological process transmit an epidemiologic effect?	Causal mediation analysis
Potential residual confounding	Could the observed association arise from an unmeasured factor?	Negative‐control designs/sensitivity analysis
Phenotypic heterogeneity	Do distinct clinical trajectories reflect different mechanisms?	Latent class/mixture models
Effect modification	Does susceptibility differ by genotype, sex, age, or exposure context?	Interaction modelling
Environmental contribution	Which exposure mixtures correspond to population structure?	Exposure reconstruction/exposome approaches

Potential methodological approaches include:

- longitudinal life‐course cohorts,
- migration studies,
- multimorbidity network analysis,
- latent class modelling,
- causal mediation analysis,
- negative‐control designs,
- explicit modelling of interaction terms,
- integration of environmental exposure reconstruction [6, 7, 8, 32, 33, 34, 35, 36].

The framework also supports incorporation of resilient populations as potentially informative models of regulatory protection [29, 30, 31].

Rather than treating epidemiologic heterogeneity primarily as statistical noise, this approach evaluates heterogeneity as potentially informative regarding mechanistic organization. The proposed inferential sequence is summarised in Figure 1.

**Figure 1:**
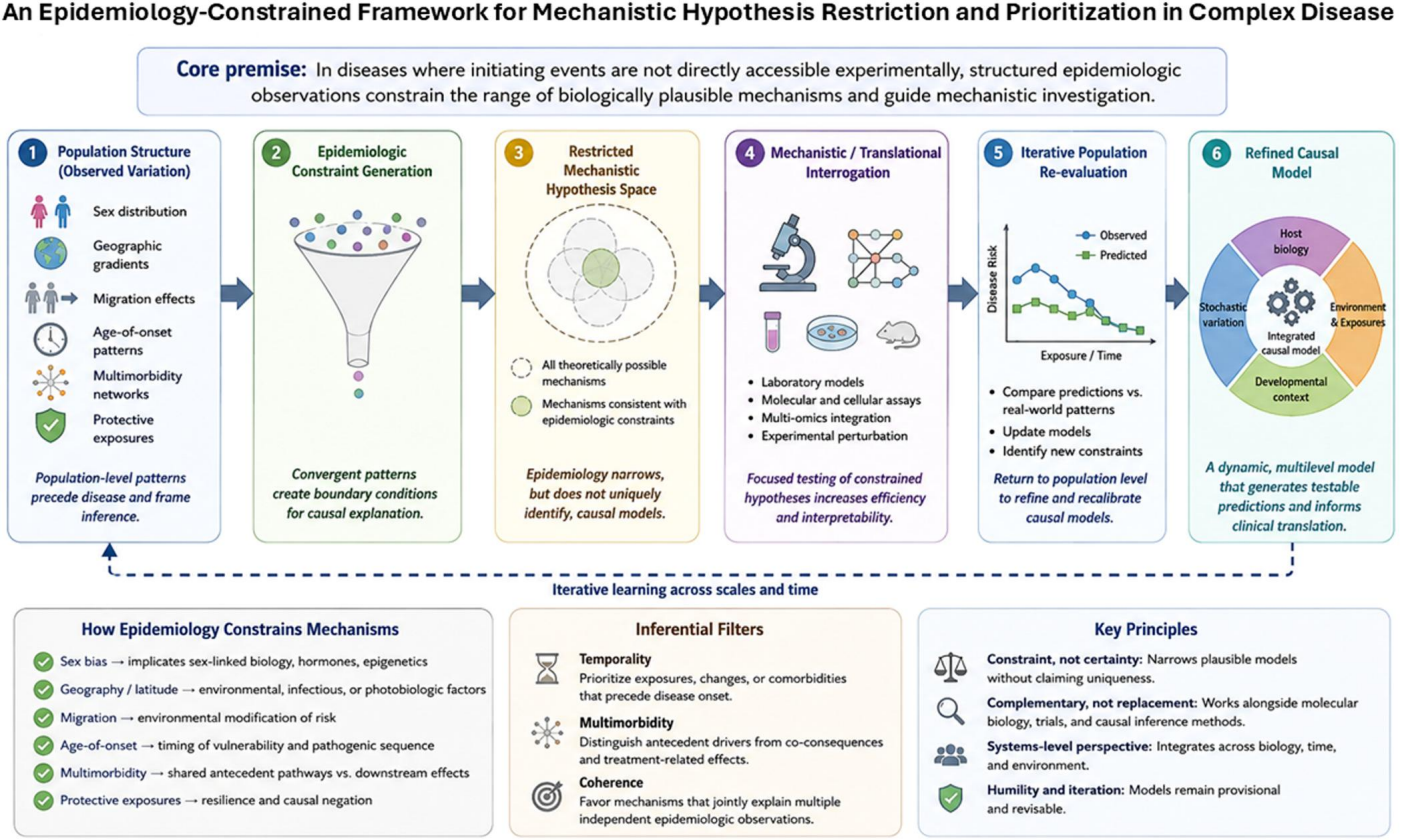
An epidemiology‐constrained framework for mechanistic hypothesis restriction and prioritization in complex disease.

This perspective is also supported by historical precedent. Convergent epidemiologic structure has frequently preceded mechanistic clarification, including smoking‐associated lung cancer, developmental origins of disease and infectious contributions to peptic ulcer disease. Epidemiology‐constrained inference may fail when population‐level structure reflects ascertainment artifacts, diagnostic instability, secular environmental change, or latent disease heterogeneity that obscures mechanistic coherence. The framework therefore depends upon reproducibility across independent populations and continual re‐evaluation as epidemiologic structure evolves.

Selection of study design should be driven not only by the observed constraint but also by the principal alternative explanation that must be tested, the anticipated source of bias, the temporal structure of the hypothesis and the degree of independence between available observations.

The purpose of this mapping is not to prescribe a single analytic method for each epidemiologic pattern, but to link the type of constraint under investigation to study designs capable of testing alternative explanations.

## 14 Limitations

Several limitations and safeguards are integral to the framework.

First, epidemiologic patterns may reflect residual confounding, ascertainment bias, healthcare‐access differences, diagnostic drift, or secular trends. Structured multimorbidity may arise partly from shared healthcare utilization rather than shared biology.

Second, epidemiology‐constrained inference does not independently establish causality. The framework is intended to prioritise and restrict mechanistic hypotheses under conditions of incomplete experimental accessibility rather than replace experimental validation.

Third, multiple mechanistic models may remain compatible with the same epidemiologic structure. Constrained inference narrows hypothesis space but does not necessarily produce unique causal explanations. Importantly, epidemiologic coherence does not guarantee mechanistic correctness, since distinct biological architectures may generate similar population‐level patterns. Furthermore, some apparently antecedent epidemiologic observations may themselves represent early manifestations of subclinical disease processes. Accordingly, epidemiologic structure may constrain mechanistic plausibility without uniquely establishing causal directionality.

Fourth, there is risk of retrospective coherence in which observed epidemiologic patterns appear more mechanistically unified than they truly are.

A related limitation is confirmation bias within the framework itself. Once a mechanistic model becomes favoured, investigators may selectively interpret epidemiologic observations as supporting constraints. To reduce this risk, candidate constraints should, where feasible, be prespecified before mechanistic testing, disconfirming observations should be actively sought, and competing mechanistic models should be evaluated using the same constraint set. Independent replication of the epidemiologic structure provides an additional safeguard against model‐dependent interpretation. Where independent replication is unavailable, conclusions should be reported explicitly as provisional and sensitive to the limitations of the available population structure.

Finally, different diseases may vary substantially in the extent to which epidemiologic structure meaningfully constrains mechanistic interpretation.

The framework is also dynamic rather than static. Population‐level patterns may change because of diagnostic practices, screening, treatment, vaccination, environmental change, migration, or population aging. Accordingly, stable mechanistic constraints should be distinguished from historically contingent epidemiologic patterns. Re‐evaluation across time and populations is therefore necessary before a population pattern is treated as a durable boundary condition on mechanistic models.

## 15 Conclusion

Complex diseases characterised by prolonged latency and multiscale interaction often resist direct experimental reconstruction of initiating causal events. Under such circumstances, epidemiologic structure may provide an important source of mechanistic constraint.

Sex distribution, geographic gradients, migration effects, age‐of‐onset patterns, multimorbidity structure and protective exposures are not solely descriptive observations; collectively, they may help restrict the range of biologically plausible mechanisms compatible with disease emergence.

We propose that epidemiology‐constrained mechanistic hypothesis restriction and prioritization offers a complementary framework for organising hypothesis generation in complex chronic disease. The framework does not establish mechanisms or causality independently. Rather, it uses reproducible, bias‐assessed, and where feasible independently replicated epidemiologic structure to restrict competing mechanistic models and prioritise those that remain compatible with population‐level evidence for subsequent molecular, experimental and translational testing.

Schematic overview of the proposed framework in which structured epidemiologic observations function as inferential constraints on mechanistic hypothesis generation under conditions of limited experimental accessibility. Population‐level patterns, including sex distribution, geographic gradients, migration effects, age‐of‐onset structure, multimorbidity networks and protective exposures, are conceptualised as upstream epidemiologic constraints that narrow the range of biologically plausible mechanisms compatible with disease emergence. These constraints restrict the mechanistic hypothesis space prior to molecular and translational interrogation, thereby guiding experimental prioritization. Iterative comparison between experimentally derived models and real‐world population structure permits refinement and prioritization of mechanistic hypotheses over time, without independently establishing causality. The framework emphasises that epidemiologic observations do not independently establish causality, but may help organise mechanistic hypothesis restriction and prioritization in diseases characterised by prolonged latency, developmental susceptibility windows and multiscale biological interaction. Temporality, multimorbidity structure, and coherence across independent observations function as inferential filters within the model. Mechanistic models inconsistent with stable epidemiologic structure may undergo iterative revision or deprioritization.

## Author Contributions

Uri Gabbay conceived the study, developed the conceptual framework, performed the literature review, interpreted the relevant evidence, draughted the manuscript, and approved the final version for submission.

## Funding

The authors have nothing to report.

## Ethics Statement

Ethical approval was not required for this study because it is a conceptual analysis based exclusively on published literature and did not involve human participants, animals, identifiable personal data, or primary data collection.

## Conflicts of Interest

The author declares no conflicts of interest.

## Data Availability

No new data was generated or analysed in support of this conceptual article. Data sharing is therefore not applicable to this study.

## References

[1] G. Frazzei , R. F. van Vollenhoven , B. A. de Jong , S. E. Siegelaar , and D. van Schaardenburg , “Preclinical Autoimmune Disease: A Comparison of Rheumatoid Arthritis, Systemic Lupus Erythematosus, Multiple Sclerosis and Type 1 Diabetes,” Frontiers in Immunology 13 (2022): 899372. 10.3389/fimmu.2022.899372 PMC9281565 35844538

[2] M. Wahren‐Herlenius and T. Dörner , “Immunopathogenic Mechanisms of Systemic Autoimmune Disease,” Lancet 382, no. 9894 (2013): 819–831. 10.1016/S0140-6736(13)60954-X 23993191

[3] F. W. Miller , “Environment, Lifestyles, and Climate Change: The Many Nongenetic Contributors to the Long and Winding Road to Autoimmune Diseases,” Arthritis Care & Research 77, no. 1 (2025): 3–11. 10.1002/acr.25423 PMC11684977 39228044

[4] N. Petrovská , K. Prajzlerová , J. Vencovský , L. Šenolt , and M. Filková , “The Pre‐Clinical Phase of Rheumatoid Arthritis: From Risk Factors to Prevention of Arthritis,” Autoimmunity Reviews 20, no. 5 (2021): 102797. 10.1016/j.autrev.2021.102797 33746022

[5] K. Donald and B. B. Finlay , “Early‐Life Interactions Between the Microbiota and Immune System,” Nature Reviews Immunology 23, no. 11 (2023): 735–748. 10.1038/s41577-023-00874-w 37138015

[6] W. J. Gauderman , B. Mukherjee , H. Aschard , et al., “Update on the State of the Science for Analytical Methods for Gene‐Environment Interactions,” American Journal of Epidemiology 186, no. 7 (2017): 762–770. 10.1093/aje/kwx228 PMC5859988 28978192

[7] K. McAllister , L. E. Mechanic , C. Amos , et al., “Current Challenges and New Opportunities for Gene‐Environment Interaction Studies,” American Journal of Epidemiology 186, no. 7 (2017): 753–761. 10.1093/aje/kwx227 PMC5860428 28978193

[8] A. T. DeWan , “Gene‐Gene and Gene‐Environment Interactions,” Methods in Molecular Biology 1793 (2018): 89–110. 10.1007/978-1-4939-7868-7_7 29876893

[9] D. Fairweather , D. J. Beetler , E. J. McCabe , and S. M. Lieberman , “Mechanisms Underlying Sex Differences in Autoimmunity,” Journal of Clinical Investigation 134, no. 18 (2024): e180076. 10.1172/JCI180076 PMC11405048 39286970

[10] D. Bartz , T. Chitnis , U. B. Kaiser , et al., “Clinical Advances in Sex‐ and Gender‐Informed Medicine,” JAMA Internal Medicine 180, no. 4 (2020): 574–583. 10.1001/jamainternmed.2019.7194 32040165

[11] K. C. Dodd and M. Menon , “Sex Bias in Lymphocytes: Implications for Autoimmune Diseases,” Frontiers in Immunology 13 (2022): 945762. 10.3389/fimmu.2022.945762 PMC9730535 36505451

[12] R. Shepherd , A. S. Cheung , K. Pang , et al., “Sexual Dimorphism in Innate Immunity,” Frontiers in Immunology 11 (2020): 604000. 10.3389/fimmu.2020.604000 PMC7873844 33584674

[13] S. Berrih‐Aknin , R. L. Panse , and N. Dragin , “AIRE and Female Susceptibility to Autoimmune Disease,” Annals of the New York Academy of Sciences 1412, no. 1 (2018): 21–32. 10.1111/nyas.13529 29291257

[14] T. J. Nowak and M. P. Muehlenbein , “Toward Understanding Sexual Immune Dimorphism in Humans,” Frontiers in Immunology 16 (2025): 1570565. 10.3389/fimmu.2025.1570565 PMC12226279 40621451

[15] C. E. Sabel , J. F. Pearson , D. F. Mason , E. Willoughby , D. A. Abernethy , and B. V. Taylor , “The Latitude Gradient for Multiple Sclerosis Prevalence Is Established in the Early Life Course,” Brain 144, no. 7 (2021): 2038–2046. 10.1093/brain/awab104 33704407

[16] S. Simpson , W. Wang , P. Otahal , L. Blizzard , I. A. F. van der Mei , and B. V. Taylor , “Latitude Continues to Be Significantly Associated with the Prevalence of Multiple Sclerosis: An Updated Meta‐Analysis,” Journal of Neurology, Neurosurgery & Psychiatry 90, no. 11 (2019): 1193–1200. 10.1136/jnnp-2018-320189 31217172

[17] M. Ghareghani , R. J. Reiter , K. Zibara , and N. Farhadi , “Latitude, Vitamin D, Melatonin, and Gut Microbiota Act in Concert to Initiate Multiple Sclerosis: A New Mechanistic Pathway,” Frontiers in Immunology 9 (2018): 2484. 10.3389/fimmu.2018.02484 PMC6232868 30459766

[18] J. F. Bach , “Revisiting the Hygiene Hypothesis in the Context of Autoimmunity,” Frontiers in Immunology 11 (2020): 615192. 10.3389/fimmu.2020.615192 PMC7876226 33584703

[19] M. F. Khan and H. Wang , “Environmental Exposures and Autoimmune Diseases,” Frontiers in Immunology 10 (2019): 3094. 10.3389/fimmu.2019.03094 PMC6970196 31998327

[20] Y. Shapira , N. Agmon‐Levin , and Y. Shoenfeld , “Defining and Analyzing Geoepidemiology and Human Autoimmunity,” Journal of Autoimmunity 34, no. 3 (2010): J168–J177. 10.1016/j.jaut.2009.11.018 20034761

[21] J. F. Bach , “The Effect of Infections on Susceptibility to Autoimmune and Allergic Diseases,” New England Journal of Medicine 347, no. 12 (2002): 911–920. 10.1056/NEJMra020100 12239261

[22] H. Okada , C. Kuhn , H. Feillet , and J. F. Bach , “The Hygiene Hypothesis for Autoimmune and Allergic Diseases: An Update,” Clinical and Experimental Immunology 160, no. 1 (2010): 1–9. 10.1111/j.1365-2249.2010.04139.x PMC2841828 20415844

[23] D. S. Reich , C. F. Lucchinetti , and P. A. Calabresi , “Multiple Sclerosis,” New England Journal of Medicine 378, no. 2 (2018): 169–180. 10.1056/NEJMra1401483 PMC6942519 29320652

[24] M. P. McGinley , C. H. Goldschmidt , and A. D. Rae‐Grant , “Diagnosis and Treatment of Multiple Sclerosis,” Journal of the American Medical Association 325, no. 8 (2021): 765–779. 10.1001/jama.2020.26858 33620411

[25] A. B. Hill , “The Environment and Disease: Association or Causation?,” Proceedings of the Royal Society of Medicine 58 (1965): 295–300. 10.1177/003591576505800503 PMC1898525 14283879

[26] K. Barnett , S. W. Mercer , M. Norbury , G. Watt , S. Wyke , and B. Guthrie , “Epidemiology of Multimorbidity,” Lancet 380, no. 9836 (2012): 37–43. 10.1016/S0140-6736(12)60240-2 22579043

[27] C. Violan , Q. Foguet‐Boreu , G. Flores‐Mateo , et al., “Prevalence, Determinants and Patterns of Multimorbidity,” PLoS One 9, no. 2 (2014): e102149. 10.1371/journal.pone.0102149 PMC4105594 25048354

[28] J. M. Valderas , B. Starfield , B. Sibbald , C. Salisbury , and M. Roland , “Defining Comorbidity,” Annals of Family Medicine 7, no. 4 (2009): 357–363. 10.1370/afm.983 PMC2713155 19597174

[29] J. F. Bach , “The Hygiene Hypothesis in Autoimmunity: The Role of Pathogens and Commensals,” Nature Reviews Immunology 18, no. 2 (2018): 105–120. 10.1038/nri.2017.111 29034905

[30] G. Murdaca , M. Greco , M. Borro , and S. Gangemi , “Hygiene Hypothesis and Autoimmune Diseases,” Autoimmunity Reviews 20, no. 7 (2021): 102845. 10.1016/j.autrev.2021.102845 33971339

[31] M. Harel and Y. Shoenfeld , “Predicting and Preventing Autoimmunity,” Annals of the New York Academy of Sciences 1069 (2006): 322–345. 10.1196/annals.1351.031 16855160

[32] C. P. Wild , “Complementing the Genome with an ‘Exposome’: The Outstanding Challenge of Environmental Exposure Measurement in Molecular Epidemiology,” Cancer Epidemiology, Biomarkers & Prevention 14, no. 8 (2005): 1847–1850. 10.1158/1055-9965.EPI-05-0456 16103423

[33] S. Galea and K. M. Keyes , Population Health Science. Oxford University Press, 2019.

[34] K. Hoffman and S. T. Roussos , “Systems Epidemiology and Population Health,” Preventing Chronic Disease 10 (2013): E89.

[35] Y. Ben‐Shlomo , “A Life Course Approach to Chronic Disease Epidemiology,” International Journal of Epidemiology 31, no. 2 (2002): 285–293. 11980781

[36] D. A. Lawlor , K. Tilling , and G. Davey Smith , “Triangulation in Aetiological Epidemiology,” International Journal of Epidemiology 45, no. 6 (2016): 1866–1886. 10.1093/ije/dyw314 PMC5841843 28108528

